# *Eruca sativa* Might Influence the Growth, Survival under Simulated Gastrointestinal Conditions and Some Biological Features of *Lactobacillus acidophilus, Lactobacillus plantarum* and *Lactobacillus rhamnosus* Strains

**DOI:** 10.3390/ijms151017790

**Published:** 2014-10-01

**Authors:** Florinda Fratianni, Selenia Pepe, Federica Cardinale, Tiziana Granese, Autilia Cozzolino, Raffaele Coppola, Filomena Nazzaro

**Affiliations:** Institute of Food Science, ISA-CNR, Via Roma, 64, 83100 Avellino, Italy; E-Mails: fratianni@isa.cnr.it (F.F.); selenia.pepe@hotmail.it (S.P.); f.sweetgirl@hotmail.it (F.C.); tizianag46@hotmail.it (T.G.); ilia.cozzolino@hotmail.it (A.C.); coppola@isa.cnr.it (R.C.)

**Keywords:** probiotics, *Eruca sativa*, *Lactobacillus*, antioxidant, antimicrobial activity, lab-on-chip protein profile

## Abstract

The growth and viability of three *Lactobacillus* strains, *Lactobacillus acidophilus*, *Lactobacillus plantarum* and *Lactobacillus rhamnosus*, after their passage through simulated gastric and pancreatic juices were studied as a function of their presence in the growth medium of rocket salad (*Eruca sativa*). The presence of *E. sativa* affected some of the biological properties of the strains. For example, *L. acidophilus* and *L. plantarum* worked more efficiently in the presence of *E. sativa*, increasing not only the antioxidant activity of the medium, but also their own antioxidant power and antimicrobial activity; *L. rhamnosus* was not affected in the same manner. Overall, the presence of vegetables might help to boost, in specific cases, some of the characteristics of lactobacilli, including antioxidant and antimicrobial power.

## 1. Introduction

Fresh fruit and vegetables represent a key source of naturally occurring antioxidants. It has been reported that a diet containing plant antioxidants may reduce the risk of several diseases [1], such as cardiovascular diseases and different types of cancer, and can generally limit or prevent damage occurring to cellular components due to oxidation [2]. Among natural antioxidants, polyphenols play a very important role [3]. Many of their beneficial health effects can be ascribed to their antioxidant and chelating abilities that give rise to their ability to transfer electron-free radicals, chelate metal catalysts, activate antioxidant enzymes, reduce *R*-tocopherol radicals and inhibit oxidases [4]. Rocket salad (*Eruca sativa* Miller) is an annual herb belonging to the Brassicaceae (or Cruciferae) family. It is widely recognized to be an important ingredient for culinary and medical/herbalist purposes. Similarly to many of the other Brassicaceae, *Eruca sativa* consumption might be associated with a highly significant reduction in cancer [5]. *Eruca sativa* has been known since ancient times in the Mediterranean area and in Central to Western Asia. At present, it is cultivated in Northern Europe, northern America and India. Leaves of rocket salad are intensely green with notched edges and have a characteristic spicy taste that is affected by environmental conditions. Rocked salad is well-known to be a healthy plant that aids in digestion, acts as a diuretic and protects the liver. In addition, it can exhibit chemo-protective action [6], as well as impart beneficial effects on microbiota, and subsequently, it can limit the production of intestine gas. Its main beneficial properties can be ascribed to the presence of vitamin C and some mineral salts (such as iron, calcium and phosphorous) and other biomolecules, such as polyphenols. There is also some evidence that polyphenols may influence microbiota [7]. In an *in vitro* study, the isoflavone, genistein, was shown to inhibit the internalization of human Caco-2 and HT-29 cells by intestinal bacteria [8]. Parkar *et al.* [9] highlighted the potential influence of well-known polyphenols on gut micro-ecology by studying their effects *in vitro* on the growth of bacteria commonly present in the human GI tract. In particular, they reported the novel effect of polyphenols influencing the adhesion of two representative gut bacteria, *Salmonella typhimurium* (Gram-negative pathogen) and *Lactobacillus rhamnosus* GG (Gram-positive probiotic), to cultured human Caco-2 enterocytes. Extensive research into the interactions between phenolic compounds and intestinal bacteria has mainly focused on antimicrobial properties against pathogenic bacteria [10,11,12,13] to evaluate their potential effects on the shelf life of foods [14]. The growth of different lactobacilli has been previously investigated using different polyphenol matrices [15,16,17,18,19]; however, to our knowledge, there is no literature reporting the effect of the presence of *Eruca sativa* extracts on *Lactobacillus* strains. Therefore, our study aimed to evaluate the behavior of three *Lactobacillus* strains grown in the presence of *Eruca sativa*. In particular, the growth, the resistance to a simulated gastro-intestinal transit, polyphenol content and the antioxidant and antimicrobial activities, as well as total protein profiles of bacteria were evaluated.

## 2. Results and Discussion

### 2.1. Growth of Bacteria and Resistance to the Simulated Digestion

Generally, the presence of plant extracts does not affect the growth of microorganisms [20,21], but in some cases, they can benefit from the presence of these bio-components, increasing, for example, their survival during gastro-intestinal transit. This effect is influenced by both the type of strain and by the vegetable matrix [22]. Table 1 shows the growth of lactobacilli in the presence and absence of *E. sativa*, before and after simulated digestion. The data are reported as colony forming units (cfu)/mL.

**Table 1:** Viability (expressed as cfu/mL) of *L. acidophilus* (LA), *L. plantarum* (LP) and *L. rhamnosus* (LR) grown in MRS medium (see materials and methods) without (used as the control) or with *E. sativa* (+E), before the *in vitro* gastric and pancreatic transit (BGPJ and AGPJ, respectively). The data are the mean (±standard deviation) of three independent experiments.

	*T*_0_ cfu/mL (SD)	*T* 24 h BGPJ treatment cfu/mL (SD)	*T* 24 h AGPJ treatment cfu/mL (SD)
LA	1.23 × 10^2^ (0.01 × 10^2^)	1.25 × 10^8^ (0.02 × 10^2^)	1.12 × 10^5^ (0.03 × 10^2^)
LA + E	1.24 × 10^2^ (0.02 × 10^2^)	8.15 × 10^7^ (0.04 × 10^3^)	5.29 × 10^6^ (0.02 × 10^3^)
LP	1.34 × 10^2^ (0.02 × 10^2^)	1.55 × 10^8^ (0.05 × 10^3^)	2.49 × 10^8^ (0.03 × 10^3^)
LP + E	1.36 × 10^2^ (0.01 × 10^2^)	2.46 ×10^8^ (0.03 × 10^3^)	3.34 × 10^8^ (0.05 × 10^3^)
LR	1.27 × 10^2^ (0.02 × 10^2^)	1.14 × 10^8^ (0.02 × 10^3^)	1.91 × 10^7^ (0.03 × 10^3^)
LR + E	1.25 × 10^2^ (0.02 × 10^2^)	4.43 × 10^7^ (0.05 × 10^3^)	4.16 × 10^6^ (0.03 × 10^3^)

*L. acidophilus* exhibited just a slightly less effective capability to grow in the presence of *E. sativa* than the control (containing 1.25 × 10^8^ cfu/mL). Additionally, the presence of *E. sativa* seemed to “protect” the microorganism during the simulated digestive process. The *L. acidophilus* grown in medium MRS showed a marked decrease of cfu/mL from 1.25 × 10^8^ to 1.12 × 10^5^; however, in the presence of the extract, it exhibited a greater resilience to the adverse simulated environmental conditions, so that its viability changed from 8.15 × 10^7^ cfu/mL to 5.29 × 10^6^ cfu/mL, a value still acceptable in terms of probioticity (1 × 10^6^–1 × 10^8^ cfu/mL, as reported by the FAO in 2001) [23]. *L. plantarum* was not positively influenced by the presence of polyphenols; however, these increased its capability to resist stress. An opposite behavior was demonstrated by *L. rhamnosus*, which did not receive a clear benefit from the presence of polyphenols in the culture medium before or after the simulated digestion. The presence of polyphenols seemed to dampen bacterial growth compared with the control, and taking into consideration only this parameter, *E. sativa* could not affect the growth or the resistance to gastro-intestinal transit in any way.

### 2.2. Total Polyphenols and Antioxidant Activity

Some dietary products have a well-known potential to improve the growth and beneficial activities of lactobacilli [24], for example metabolites, such as glycosides and flavonoids [25,26]. In our experiments, a dosage of total polyphenols was transferred to culture medium, with and without *E. sativa*, to have two comparison values and to assess whether any changes with respect to the content of polyphenols could be attributed to the presence of lactobacilli. The results shown in Table 2 are expressed in µg/mL, compared with the equivalent weight of gallic acid (GAE) used as a standard.

**Table 2:** Total polyphenols present in MRS medium with and without *E. sativa*, before and after 24 h of growth of *L. acidophilus* (LA), *L. plantarum* (LP) and *L. rhamnosus* (LR). The data are expressed as µg gallic acid equivalent (GAE)/mL and represent the mean (±standard deviation) of three independent experiments.

	Total polyphenols	
	*T* = 0 μg GAE/mL (SD)	*T* = 24 h μg GAE/mL (SD)
MRS	130.36 (1.07)	-
MRS + *E.sativa*	230.48 (2.21)	-
LA	-	139.25 (2.55)
LA + *E.sativa*	-	218.14 (5.14)
LP	-	138.04(2.12)
LP + *E. sativa*	-	143.62 (3.01)
LR	-	136.06 (0.55)
LR + *E. sativa*	-	204.03 (1.43)

At time zero, total polyphenols (TPF) in MRS were 130 µg GAE/mL. The presence of *E. sativa* increased the value by nearly 100% (230 µg GAE/mL). The growth of *L. acidophilus* in MRS did not influence the content of the total polyphenols of the medium. A decrease of approximately 5% (218 μg/mL *versus* 230 μg/mL, respectively) was detected when the strain grew in MRS broth containing *E. sativa*. *L. rhamnosus* exhibited a similar behavior, and a decrease of 10% was observed when it was grown in the presence of *E. sativa. L. plantarum* exhibited a totally different behavior when grown in the presence of *E. sativa*; it apparently “consumed” 38% of the TPF compared to the unfermented medium (143 µg GAE/mL *versus* 230 µg GAE/mL, respectively). The determination of the antioxidant potential of polyphenols was performed using the DPPH test, both on the medium and on microbial pellets, after 24 h of fermentation. This analysis was carried out to determine whether the presence of *E. sativa* could affect the antioxidant activity of the bacteria compared to the standard conditions. The results shown in Table 3 are expressed, with regard to the medium of growth, as EC_50_, indicating the volume of sample (µL) needed to decrease the power of 1 mL of the stable radical DPPH by 50% and in the percentage of activity for the bacterial pellet.

**Table 3:** Antioxidant activity evaluated using a DPPH test of medium and microbial pellets of *L. acidophilus* (LA), *L. plantarum* (LP) and *L. rhamnosus* (LR) grown in MRS or MRS + *E. sativa*. The data are expressed in terms of EC_50_, the amount (as µL) of sample needed to decrease the activity of 1 mL of the stable radical DPPH by 50%. The data represent the mean (±standard deviation) of three independent experiments.

	DDPPH test on growth medium		DDPPH test on microbial pellet
	*T* = 0	*T* = 24 h	*T* = 24 h
	EC_50_ (μL/mL ± SD)	EC_50_ (μL/mL ± SD)	Antioxidant power (%)
MRS	74.73 (2.64)		
MRS + *E.sativa*	49.29 (2.37)		
LA		92.13 (5.99)	17.90
LA + *E.sativa*		48.64 (1.15)	22.95
LP		84.80 (6.69)	19.41
LP + *E.sativa*		63.98 (1.98)	24.02
LR		70.38 (2.53)	15.23
LR + *E.sativa*		51.14 (2.39)	14.03

The presence of *E. sativa* obviously increased the antioxidant activity of the medium compared to the control: 25 µL less (49.29 µL *versus* 74.73 µL) of the culture medium than of the control were needed to inhibit the activity of 1 mL of the DPPH radical by 50%. In the absence of *E. sativa*, *L. acidophilus* failed to decrease the volume of broth needed to lead to a 50% decrease in the activity of 1 mL of DPPH: EC_50_ increased from 74.73 at time zero to 92.13 µL after 24 h. In the presence of *E. sativa*, *L. acidophilus* did not affect the initial antioxidant activity. Indeed, the antioxidant power exhibited by its pellet increased by 5% if compared to the standard growth conditions, demonstrating that the presence of the vegetable in the culture medium and the concurrent growth of this strain did not give a lowering of the antioxidant activity of the medium; indeed, such a situation might beneficially impact the antioxidant activity of the strain, which could bring further positive effects, such as higher antioxidant activity, when grown under these conditions (for example, in the presence of fruits and vegetables) and then could be an additional means to counteract the action of free radicals. The presence of *L. plantarum* in the medium containing *E. sativa* mitigated, albeit more weakly than *L. acidophilus*, the loss of the antioxidant activity of the medium itself (EC_50_ = 63 µL). Furthermore, in this case, the presence of *E. sativa* gave rise to an increase of the anti-radical properties of the strain, which pellet increased its antioxidant power from 19% to approximately 24%, in line with that exhibited by *L. acidophilus*. A quite different behavior was exhibited by *L. rhamnosus*. Although its presence did not affect the antioxidant activity of medium containing *E. sativa*, the strain was not positively affected by its own antioxidant activity; thus, after 24 h of growth, the percentage remained virtually the same (14%) as that observed in standard conditions of growth (15%). This could support the idea that bacteria have a metabolism that can be expressed in different ways and in a different manners depending on the presence of diverse components [20].

### 2.3. Antimicrobial Activity

Phytochemicals are able to change some beneficial probiotic species while inhibiting the growth of non-beneficial species, such as *Bacteroides*, *Clostridia*, *Coliforms* and *Salmonella* spp. [27,28]. The antimicrobial power exhibited by the three *Lactobacillus* after 24 h of growth, before and after the digestive passage, was measured using the inhibition halo test. The results are shown in Table 4. In Table 5, pathogen sensitivity to DMSO, MRS, MRS + *E. sativa* and tetracycline are shown.

The presence of polyphenols in the culture medium might have transformed bacterial metabolism leading also to the production of substances with antimicrobial activity. *L. plantarum* grown in MRS demonstrated antimicrobial activity against all pathogenic strains used as standards, with halos of inhibition ranging between 2 and 12 mm (Table 4, green). In some case, the simulation of the gastro-intestinal transit demonstrated a slight decrease in the antimicrobial ability of the strain grown in MRS, in particular against *E. coli* (with a halo of inhibition of only 3 mm, with the highest volume of culture supernatant used in our experiment, compared to a halo of 9 mm, observed in the pre-gastric situation). The two strains of *B. cereus* used as standards showed slightly different behaviors, mainly after the simulated digestion. This suggests that for antioxidant activity, the presence of phytochemicals might affect the reaction of the microorganisms, even between two strains of the same species. For this reason, testing multiple strains of the same species in the evaluation of antimicrobial activities of any biological matrix (cells or natural extracts) should be considered. De Martino *et al.* [29], for example, observed a different behavior between two strains of *B. cereus* placed in contact with different essential oils and noted that *B. cereus* 4384 was more sensitive to the action of the essential oils tested than *B. cereus* 4313. These results, despite being obtained with *Lamiaceae* and not with *Brassicaceae*, are in agreement with our data. The presence of polyphenols in the growth medium caused a change in the behavior of *L. plantarum* compared with the control, especially after the simulation of the digestive process. *L. acidophilus* grown in MRS showed antimicrobial activity against all pathogens used as tester strains (Table 4, orange). The digestive process brought about a decrease of its antimicrobial activity, so that only *P. aeruginosa* retained a certain sensitivity (albeit decreased by 50% before *in vitro* digestion) at all three concentrations used in the experiment, and *B. cereus* was sensitive at the highest concentration used in our experiments. The presence of *E. sativa* increased the antimicrobial activity of *L. acidophilus* (the effect was enhanced after the simulated digestion) against *B. cereus* 4313 and *S. aureus* (with the highest concentration).

The antimicrobial activity of *L. rhamnosus* decreased (Table 4, purple), in some cases by more than 50% after *in vitro* digestion, but the presence of *E. sativa* did not deteriorate the antimicrobial activity of the strain, especially after simulated digestion. A 75% loss of activity in the pre-digestive step (mainly against *Ps. aeruginosa* and *E. coli*) was detected.

### 2.4. Protein Profile

**Figure 1:**
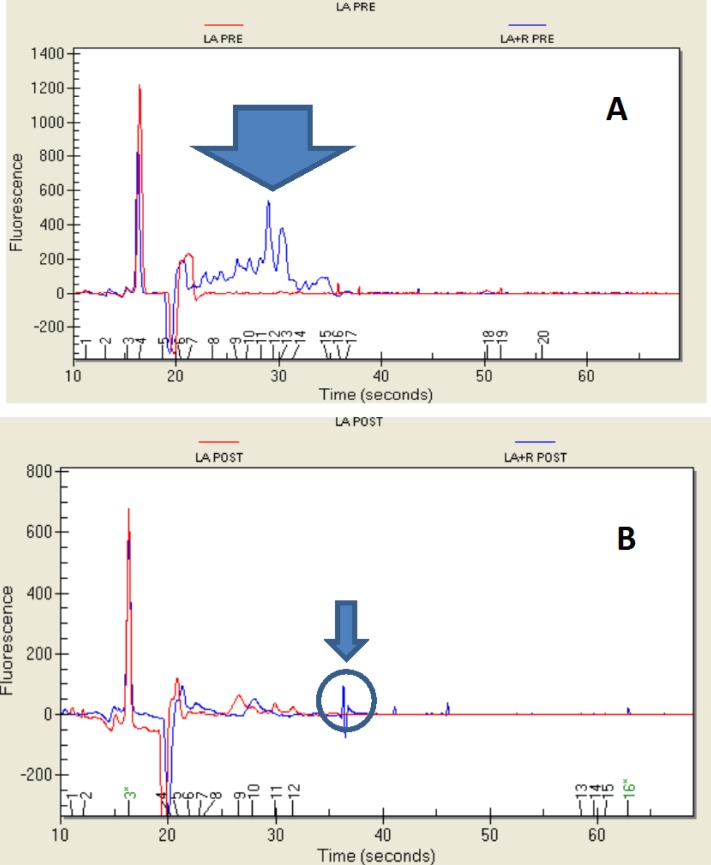
Protein profile (shown as an electropherogram) of *Lactobacillus acidophilus*, grown in MRS and MRS + *E. sativa*, before (LA PRE and LA + R PRE) (**A**) and after (LA POST and LA + R POST) (**B**) the *in vitro* gastro-intestinal transit.

**Figure 2:**
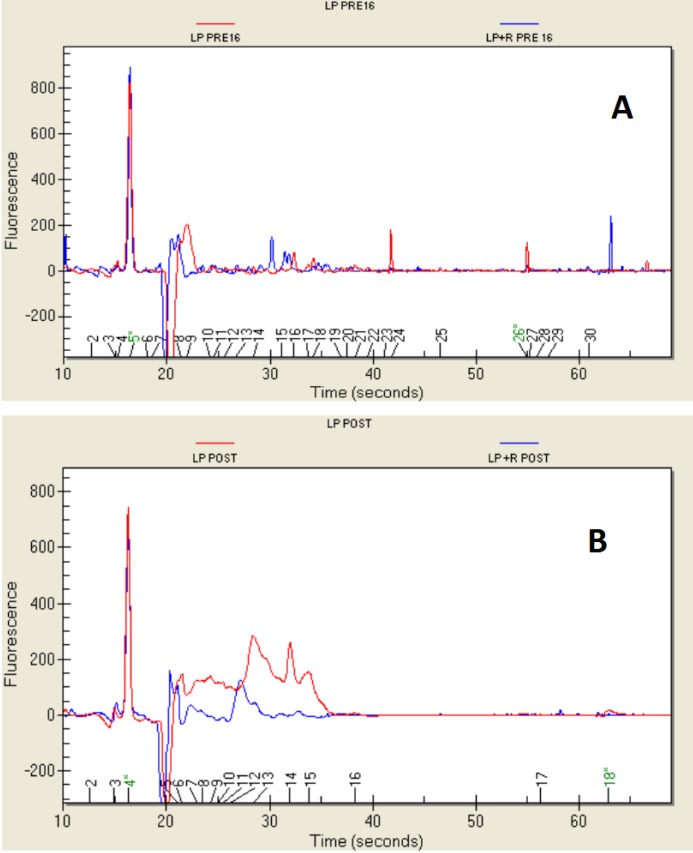
Protein profile (shown as an electropherogram) of *Lactobacillus plantarum*, grown in MRS and MRS + *E. sativa*, before (LP PRE and LP + R PRE) (**A**) and after (LP POST and LP + R POST) (**B**) the *in vitro* gastro-intestinal transit.

**Figure 3:**
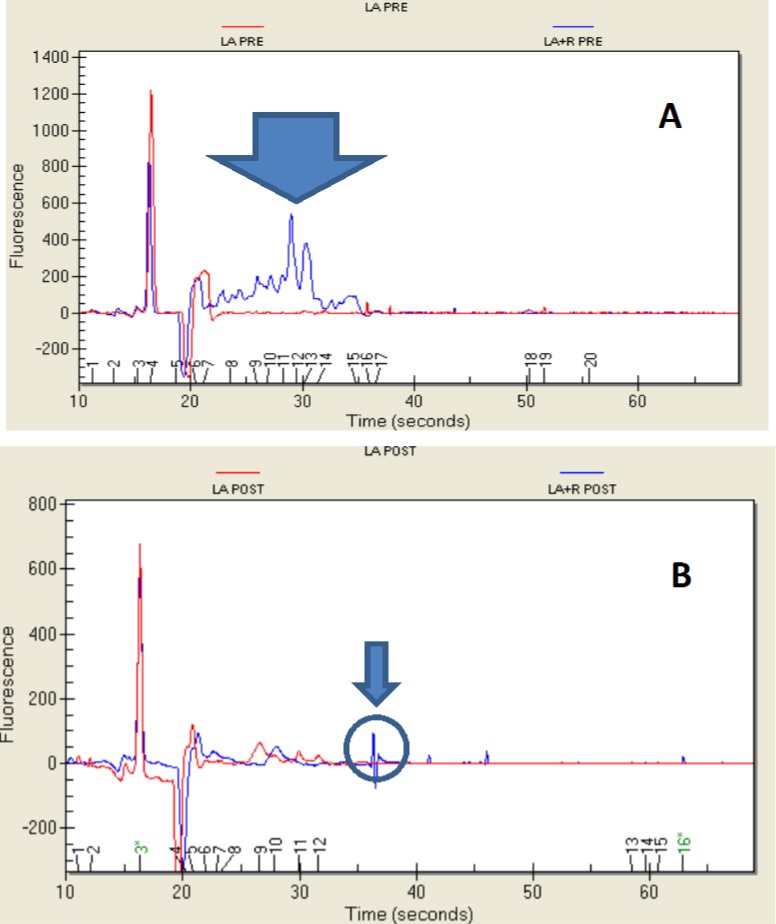
Protein profile (shown as electropherogram) of *Lactobacillus rhamnosus* grown in MRS and MRS + *E. sativa* before (LR PRE and LR + R PRE) (**A**) and after (LR POST and LR + R POST) (**B**) intestinal transit.

The analysis of the protein profile of the three lactobacilli grown in the presence and absence of extracts of *E. sativa* was performed using on-chip microelectrophoresis. The results are shown in Figure 1, Figure 2 and Figure 3. *L. acidophilus* (Figure 1) showed the greatest differences in protein patterns before the digestive *in vitro* process and with the presence or absence of *E. sativa* in the culture broth.

The two electropherograms (Figure 1A) showed large differences in protein profiles in the area ranging between 22 s and 37 s. There was an evident flattening of the peaks in the representative proteins exhibited by the strain grown in MRS in this zone. In contrast, when the *L*. *acidophilus* strain was grown in the presence of *E. sativa*, the same area showed more peaks with molecular weights ranging from 8.3 to 78.72 kDa, in particular between 24.6 and 78.7 kDa. An MW of 39.29 kDa represented 21.8% of the total proteins, and 46.93 kDa represented 19.5% of the total proteins; these were the two most common proteins in the culture despite being undetectable in the control. After the simulated digestion, the two profiles were practically similar. A protein with an MW of 97.66 kDa (marked with a circle) was observed only in the profile of the strain grown in the presence of *E. sativa* (Figure 1B), in addition to heavier proteins, MW 165 kDa and 180 kDa, present only in traces.

**Table 4:** Antimicrobial activity (evaluated through the inhibition halo test) of the filtered supernatant of MRS and MRS + *E. sativa* after the growth of *L. acidophilus* (LA, orange), *L. plantarum* (LP, green) and *L. rhamnosus* (LR, purple) before (BGPJ) and after (AGPJ) the *in vitro* gastro-intestinal transit. The results are reported as mm of halo (±standard deviation) and are the mean of three independent experiments. BC 4313: *Bacillus cereus* 4313; BC 4384: *Bacillus cereus* 4384; SA: *Staphylococcus aureus*; EC: *Escherichia coli*; PA: *Pseudomonas aeruginosa*.

	LP		LP + E		LA		LA + E		LR		LR + E	
	BGPJ	AGPJ	BGPJ	AGPJ	BGPJ	AGPJ	BGPJ	AGPJ	BGPJ	AGPJ	BGPJ	AGPJ
***BC*** **4313**												
5 µL	-	-	5 (0.5)	2 (0.3)	3.4 (0.3)	-	3 (0)	-	5 (0.3)	1.2 (0)	2 (0)	2 (0)
10 µL	4 (0.3)	-	7 (0.3)	4 (0.2)	6.3 (0.3)	3 (0.3)	6 (0.1)	4 (0)	7 (0.5)	3 (0)	6 (0.1)	3 (0)
20 µL	8 (0.8)	10 (0.4)	12 (0.2)	6 (0.3)	10.3 (0.3)	2.5 (0.5)	7 (0.6)	10.5 (0.3)	12 (0.7)	5 (0.5)	10 (0.4)	6 (0.2)
***BC* 4384**												
5 µL	2.5 (0.1)	3 (0.1)	5 (0.5)	4 (0.3)	4 (0)	-	5 (0.2)	2 (0)	4 (0.3)	2 (0.2)	3.2 (0.3)	3 (0.3)
10 µL	7 (0.3)	9 (0.3)	6 (0.4)	6 (0.2)	7 (0.3)	3 (0)	6.5 (0.5)	4 (0.1)	7.3 (0.4)	4 (0.4)	7.1 (0.4)	3 (0.3)
20 µL	12 (0.7)	12 (0.4)	11 (0.4)	12 (0.4)	9.4 (0.6)	-	10 (0.3)	10 (0.3)	11 (0.5)	6 (0.1)	11.3 (0.3)	5.9 (0.3)
***SA***												
5 µL	-	-	5 (0.5)	2 (0.2)	4.5 (0.5)	-	3.5 (0.5)	3 (0.3)	5 (0)	2 (0)	4 (0.3)	1.5 (0)
10 µL	2 (0.2)	6 (0.2)	6 (0.1)	3 (0.3)	6.5 (0.5)	3 (3.1)	6.5 (0.3)	5 (0.4)	7 (0.4)	3 (0)	6 (0.2)	3 (0)
20 µL	7 (0.4)	6 (0.3)	10 (0.3)	4 (0.2)	8.5 (0.5)	6 (0.2)	7.8 (0.3)	10 (0.3)	9 (0.3)	5 (0.5)	9 (0.3)	5 (0.5)
***PA***												
5 µL	2 (0.2)	2 (0.1)	4 (0.4)	2 (0.1)	4 (0.3)	1.5 (0.3)	4.5 (0.5)	-	-	-	2.2 (0.3)	-
10 µL	5 (0.1)	3 (0.2)	7 (0.4)	3 (0.3)	8 (0.4)	4 (0.2)	6 (0.6)	2 (0.1)	7 (0.5)	2 (0.1)	6 (0.4)	2 (0.3)
20 µL	10 (0.3)	5 (0.2)	12 (0.8)	3 (0.2)	8.5 (0.5)	4 (0.1)	9 (0.3)	2 (0.1)	11 (0.4)	3 (0.1)	10.5 (0.3)	3.2 (0.3)
***EC***												
5 µL	-	-	5 (0.2)	2 (0.1)	2.5 (0.1)	-	4.8 (0.3)	2 (0.2)	3 (0.1)	2 (0)	-	-
10 µL	2 (0.1)	-	8 (0.3)	1 (0)	7 (0.4)	3 (0.5)	6.5 (0.5)	4 (0.3)	6 (0.2)	1.3 (0)	7.5 (0.3)	-
20 µL	9 (0.8)	3 (0.3)	12 (0.3)	3 (0)	10.4 (0.1)	6 (0.1)	10 (0.3)	10 (0.6)	11 (0.4)	5 (0)	11 (0.4)	3 (0)

**Table 5:** Antimicrobial activity (evaluated through the inhibition halo test) of the filtered supernatant of MRS and MRS + *E. sativa*, DMSO (negative control) and tetracycline (7 µg, positive control) against *Bacillus cereus* 4313, *Bacillus cereus* 4384, *Staphylococcus aureus*, *Escherichia coli* and *Pseudomonas aeruginosa*.

	Gram-positive tester strains		Gram-negative tester strains		
	*P. aeruginosa*	*E. coli*	*S. aureus*	*B. cereus* *4313*	*B. cereus 4384*
DMSO	0	0	0	0	0
Tetracycline (7 μg)	9.8 (1.6)	12 (1.2)	11 (0.4)	9 (0.6)	8.4 (1.4)
MRS	0	0	0	0	0
MRS + *E. sativa*	0	0	0	0	0

*L. plantarum* exhibited proteins with an MW ranging between 9.4 and 105.66 kDa (Figure 2A). Several proteins were clear. Three of them had similar MWs (31.09 kDa, 32.3 kDa and 33.74 kDa); a fourth had an MW of 79.06 kDa, and a fifth protein had an MW of 180.8 kDa. The fifth protein was absent after growth in the presence of *E. sativa*, but in this last case*,* a protein at MW 70.6 kDa was observed that was absent in the control. When the strain was subjected to the simulated digestion, (Figure 2B), it showed three abundant proteins: MW 35.95 kDa (43% of the total), MW 58.43 kDa (12% of the total) and MW 71.4 kDa (12% of the total). The profile was slightly different when the strain grown in the presence of *E. sativa* was subjected to the simulated digestion. The first part of the electropherogram figure was more delineated than the control, and two distinct peaks were clearly visible. However, these were signals of the analytical system and were not taken into consideration. In the next portion of the electropherogram, eluted around 27 s, two enlarged peaks were quite evident at MW 29.38 kDa (60% of the total) and 36.78 kDa (12.9% of the total).

The proteins of *L. rhamnosus* grown in MRS ranged between 13 and 91 kDa (Figure 3A). Two proteins were particularly representative: MW 23.85 kDa (29% of the total proteins) and 90.45 kDa (21% of the total proteins). In the simulated pre-gastric phase (indicated as PRE in the figure), two different proteins, MW 63.56 kDa (11% of the total) and MW 92 kDa (73% of the total), were detected in the control. The presence of *E. sativa* most likely affected the microbial protein profile; the strain showed proteins at an MW ranging between 53.76 and 122.14 kDa. After simulation of the gastro-intestinal transit, many proteins were detected in the control at MWs ranging from 9.3 to 127 kDa (Figure 3B). The proteins at MW 9.42 kDa and at MW 30 kDa were the most abundant and represented 10.8% and 67% of the total protein pattern, respectively. In contrast, two proteins were observed after the growth of the strain in the presence of *E. sativa* and following the *in vitro* digestion at MW 9.30 kDa (58.8% of total) and at MW 51.17 kDa (20.11% of total).

## 3. Experimental Section

### 3.1. Strain and Culture Conditions

*Lactobacillus plantarum* subsp. *plantarum* DSM 20174, *Lactobacillus acidophilus* DSM 20079, and *Lactobacillus rhamnosus* DSM 20711 are three DSMZ reference strains (Deutsche Sammlung von Mikroorganismen und Zellkulturen GmbH, Braunschweig, DSMZ, Germany). Cells were anaerobically grown at 30 °C (*L. plantarum*) and at 37 °C (*L. acidophilus* and *L. rhamnosus*) for 24 h in MRS (de Man, Rogosa and Sharpe) broth purchased by Sigma Aldrich Italia (Milano, Italy) [30]. Gram-positive *Bacillus cereus* (strains DSM 4313 and DSM 4384) and *Staphylococcus aureus* DSM 25923 and Gram-negative *Escherichia coli* DSM 8579 and *Pseudomonas aeruginosa* ATCC 50071 were the pathogenic strains used for the antimicrobial test; they were obtained from the DSMZ. Each strain was incubated at 37 °C for 18 h in tryptone yeast extract (Oxoid, Milano, Italy).

### 3.2. Preparation of MRS Broth + Eruca sativa

To make the medium, 100 mL of MRS broth was mixed with 100 g of *E. sativa* (purchased at a local market in Avellino, Italy; previously cleaned and cut into small pieces). The mixture was immediately treated with 5 cycles of microwaves for 20 s/each, using a domestic microwave oven (Candy J51, purchased at a local household store) at the maximum power (indicated as “10” by the apparatus). After 1 h at 4 °C in the dark, the mixture was treated again with microwaves (at the above conditions), held for 1 h at 4 °C and filtered (0.22 μm, Millipore SpA, Milano, Italy). Aliquots of MRS + *E. sativa* and MRS alone (control) were inoculated with the three *Lactobacillus* and cultivated for 24 h in anaerobic conditions.

### 3.3. Resistance to Simulated Gastric and Intestinal Juices

The artificial gastric juice contained MRS broth plus 3 g/L pepsin (Sigma Aldrich Italia, Milano, Italy), with a final pH adjusted to 2.0 with HCl (Carlo Erba, Milano, Italy). The artificial intestinal juice contained MRS (Sigma Aldrich Italia, Milano, Italy) broth plus 1 g/L pancreatin (Sigma Aldrich Italia, Milano, Italy) and 4.5 g/L bile salts (sodium glycocholate and sodium taurocholate, code number X606C, Oxoid, Basingstoke, UK). Both solutions were filter-sterilized (0.22 μm, Millipore SpA, Milano, Italy). The strain was treated using the protocols described by De Giulio *et al.* [31]. Cells were harvested by centrifugation for 5 min at 5000*× g* at 4 °C (Biofuge, Beckmann Coulter Italia, Cassina de Pecchi, Italy), washed twice with physiological solution (0.85% NaCl, Carlo Erba, Milano, Italy) and centrifuged again. The pellet obtained was incubated in artificial gastric juice for 180 min at 37 °C and recovered by centrifugation. After washing with sterile physiological solution, the pellet was re-suspended in the simulated gastrointestinal juice (4.5 mL for 100 μL of cells). Samples were incubated at 37 °C for 60 min. Cell viability was evaluated by anaerobic culturing on MRS plates before and after the incubation of the strains in the two simulated gastrointestinal juices. Each separate experiment was performed three times.

### 3.4. Colorimetric Analysis of Polyphenols

The total phenolic content was determined using the Folin-Ciocalteu method as described by Singleton and Rossi [32]. Then, 750 µL of pure water and 50 µL of the Folin-Ciocalteu reagent (Bio-Rad, Milano, Italy) were added to 50 µL of the suitably diluted sample extract. The mixture was held for 6 min, then 100 µL of a 7% aqueous Na_2_CO_3_ (Sigma-Aldrich Italia, Milano, Italy) solution was added. After 120 min, the absorption was measured at 760 nm against water as a blank, using a Cary UV/Vis spectrophotometer (Varian, Palo Alto, CA, USA). The amount of total phenolics was expressed as µg gallic acid equivalents (GAE)/mL of extract.

### 3.5. Preparation of Heat Killed Cells

Preparation of heat killed cells (HKC) was performed following the method described by Liu *et al.* [33] and Nazzaro *et al.* [34], with some modifications. Cells were harvested by centrifugation for 5 min at 5000× *g* at 4 °C (Biofuge, Beckmann Coulter Italia, Cassina de Pecchi, Italy). The pellet was washed twice and re-suspended in phosphate buffered saline (pH 7.2) buffer (Carlo Erba, Milano, Italy), heated at 100 °C for 30 min and dried. Samples were re-suspended again at 5 mg dry cell/mL, autoclaved at 121 °C for 15 min and stored in 4 °C until the analysis of the radical scavenging capacity was performed.

### 3.6. Free Radical Scavenging Capacity

The free radical scavenging capacity was determined using the stable radical 2,2-diphenyl-1-picrylhydrazyl (DPPH) assay [35]. The analysis was performed in microplates by adding 7.5 μL of extract to 303 μL of a methanol DPPH solution (153 mM, handmade starting from DPPH (Sigma-Aldrich Italia, Milano, Italy) and methanol (Carlo Erba, Milano, Italy)). Next, the absorbance at λ = 517 nm was measured (Cary 50 MPR, Varian, Palo Alto, USA). The absorbance of DPPH without antioxidants (control sample) was used for baseline measurements. The scavenging activity was expressed as the 50% effective concentration (EC_50_), which was defined as the sample volume (µL) necessary to inhibit 1 mL of DPPH radical activity by 50% during a 60-min incubation. These experiments were performed in triplicate, and the results are expressed as the mean values ± standard deviations.

### 3.7. Protein Profile

Cells were centrifuged (5 min at 5000× *g* at 4 °C, Biofuge, Beckmann Coulter Italia, Cassina de Pecchi, Italy) and washed twice in 0.05 M Tris–HCl, pH 7.4 (1:5 *w*:*v*, handmade from Trizma base (Sigma-Aldrich Italia, Milano, Italy) and HCl (Carlo Erba, Milano, Italy). Pellets were re-suspended in 300 μL 0.05 M Tris–HCl, pH 7.4, with 2% SDS. Following the addition of glass beads, samples were constantly mixed for 5 min and then treated for 3 min at 100 °C [34,36]. Glass beads and cell debris were removed by centrifugation, and the supernatant was recovered. The protein content was evaluated according to the method reported by Bradford [37]. A 5-μL aliquot of each sample was diluted with 84 μL of ultrapure water, and then, 2 μL of sample buffer (Experion™ Pro260 kit, Bio-Rad, Hercules, CA, USA) containing 1 μL of β-mercaptoethanol (Sigma-Aldrich Italia, Milano, Italy) were added. All samples were treated at 100 °C for 3 min. A 6-μL sample was analyzed via chip capillary electrophoresis (Experion™ Pro260 Analysis Kit, Hercules, CA, USA) over a range of molecular weights from 1.2 to 260 kDa. The analysis was completed using an Experion™ automated electrophoresis system (Bio-Rad Hercules, CA, USA) and the appropriate software (fluorescence detection with a 10-mW semiconductor, laser emitting at 630 nm). The automated analysis required 30 min for a set of 10 samples.

### 3.8. Antimicrobial Assays

The inhibition halo test on agar plates was employed to investigate the antibacterial activity of the lactobacilli before and after the simulated digestive process, and the filter paper disc method was used. Microbial suspensions (1 × 10^8^ colony forming units-CFU-/mL) were uniformly spread onto the tryptone yeast extract agar plates (Ø = 90 mm dishes). Sterile Whatman No. 1 paper filter discs (Ø = 5 mm) were impregnated with 5 µL, 10 µL and 20 µL of the medium of MRS and MRS + *E. sativa* recovered after the growth of lactobacilli and filtered. Tetracycline (7 µg/disc; Sigma-Aldrich Italia, Milano, Italy) was used as positive control. DMSO (10 μL/paper disc Sigma-Aldrich Italia, Milano, Italy), MRS and MRS + *E. sativa* were used as negative controls. Plates were left for 30 min at room temperature under sterile conditions and then incubated at 37 °C for 24 h, after which the inhibition halo around the disc was measured. The experiments were performed in triplicate and averaged.

## 4. Conclusions

The presence of *E. sativa* affected some of the biological properties of the three lactobacilli. Notably, in most cases, they increased both the antioxidant activity of the medium and their own antioxidant power, enhancing one of the essential characteristics of probiotic bacteria. For example, *L.* *acidophilus* increased the consumption of polyphenols present in the medium, with a concurrent increases in the antioxidant power of the strain and in the resistance to the simulated digestive passage. *L. plantarum* increased its capability to inhibit the activity of the DPPH radical and increased its growth, albeit to a lesser extent than *L acidophilus*. The behavior of *L. rhamnosus* was very different: It did not seem to “accept” the presence of *E. sativa* in the medium, reducing both growth and antioxidant activity. With regard to the analysis of proteins, the most visible changes were observed in *L. acidophilus* with an enhanced protein profile compared with the control. This suggests that at least in the pre-digestive step, the synthesis of particular proteins by this *Lactobacillus*, grown in the presence of polyphenols, could support the improvement of its biological properties. In contrast, the simulated digestive process resulted in a flattening of differences in protein profiles so that differences in antimicrobial activity could not be ascribed to the expression of different proteins. The protein profile of *L. plantarum* showed some changes found both before and after the simulated gastro-intestinal transit compared with the control; however, such differences did not seem to influence its antimicrobial activity. *L. rhamnosus* showed only slight differences, which were practically annulled after the *in vitro* digestion (which did not have an obvious influence on the activity of the microbial strain). Overall, the presence of vegetables might help boost some of the characteristics of lactobacilli, including the antioxidant and antimicrobial power.
